# Automated vehicles and sustainability when considering rebound effects

**DOI:** 10.1371/journal.pone.0329193

**Published:** 2025-08-01

**Authors:** Peter Letmathe, Maren Paegert

**Affiliations:** School of Business and Economics, RWTH Aachen University, Aachen, Germany; Khalifa University, UNITED ARAB EMIRATES

## Abstract

While automated vehicles are expected to lower energy consumption, improve traffic flow and enhance road safety, their deployment may increase traffic volume, leading to a rebound effect. Addressing this issue, we develop a framework to assess the environmental, social and time costs of private and of shared automated vehicle usage in urban and rural areas in Germany. When comparing the status quo and automated vehicle usage, we show that 34–47% of additional traffic volume could emerge without deteriorating current conditions, depending on the area and usage concept. Shared automated vehicles in rural areas constitute the most distinct case, as they are the most beneficial for the environment and society but are the least attractive with respect to the time costs of passengers. Policymakers and system providers should strive to mitigate this dichotomy.

## Introduction

While the transport sector is responsible for almost 29% of all carbon dioxide emissions in Europe [1], its impacts also extend to noise emissions and accidents, for example. Moreover, mobility constitutes a basic need of individuals, but entails time losses due to, e.g., executing the driving task, finding a parking spot, or congestion. Thus, ensuring more sustainable, safe and accessible mobility is a central goal for the future.

It is expected that automated vehicles (AVs) will lead to positive effects, e.g., by reducing energy needs, improving traffic flow and lowering the number of accidents [2]. Also, using automated vehicles will enable passengers to engage in other activities instead of driving, so that users might accept longer travel times. This is reflected in a lower value of time (VOT), i.e., traveling is perceived as less inconvenient, as a stated preference survey shows [3]. However, modelling studies reveal that this can also result in an increased travel demand, thus raising the amount of vehicle miles traveled (VMT) [4]. In other words, a VMT increase constitutes a rebound effect, which can emerge due to lower energy or time costs as demonstrated by Taiebat et al. [5] in a microeconomic model, impairing positive effects from AV technology. Against this background, this research examines the dual nature of AV effects with regard to sustainability through a cost-based evaluation. In this context, the advantages of AV technology should be weighed against the disadvantages due to a potential increase in VMT. A central objective of this work is to determine break-even points of VMT, up to which the advantages outweigh the disadvantages.

As the first country worldwide, Germany has issued a country-wide policy that permits the deployment of AVs on public roads in designated operational areas [6]. An analysis of German mobility behavior shows that rural areas, compared to urban areas, exhibit larger travel distances and higher automobile usage so that they are characterized by a greater amount of VMT [7]. Regarding VMT, AVs are likely to lead to an increase, with the exception of ride-sharing systems [2]. We thus employ a 2x2 study design, distinguishing between privately owned AVs (PAVs) and shared AVs (SAVs), and between urban and rural areas in Germany.

**Fig 1:**
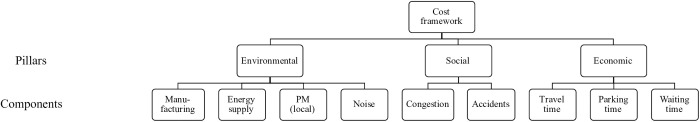
Pillars and components of the cost framework.

The impact of vehicles on the environment and society can be assessed in monetary units via external cost rates, as offered by methodological conventions from the European Commission [8] and the German Environment Agency [9]. Furthermore, we use the VOT [10] in order to assess time costs. We estimate the effects of AVs in relation to the three pillars of sustainability, regarding the environmental, social and economic perspectives (Fig 1), covering multiple dimensions as previously suggested in the literature [11,12]. The environmental pillar evaluates the emissions caused directly or indirectly by the vehicles, namely the emissions from vehicle manufacturing, the energy supply, local particulate matter (PM) and noise. There are two cost components which quantify effects that arise non-locally. First, the cost of vehicle manufacturing covers the emissions that are caused by the production of the vehicle and the factory infrastructure before the vehicle is used [9]. Second, the cost of energy supply relates to the emissions from electricity generation with regard to the German electricity mix. All other components comprise effects which occur locally from the vehicle usage. The social pillar reflects the effects that originate from the interaction of traffic participants and encompasses congestion and accident costs. These costs reflect the impact that a vehicle has on traffic reduction as well as on crash occurrence. In the economic pillar, we consider time costs. Here, travel time refers to the valuation of in-vehicle travel time, parking time covers the time spent searching for a parking space and waiting time means the time spent waiting for the arrival of a vehicle. The first two pillars describe the effects that arise for the environment and society as a whole, constituting classic externalities. The economic pillar describes costs that occur for all travelers within the regarded urban or rural areas due to time commitments. We discuss these two perspectives separately in the results.

We are not aware of any study that collectively observes environmental, social and economic effects of AV usage in relation to VMT through a cost-based evaluation in a 2x2 study design of this kind. Specifically, we address the following research questions: (1) What are the environmental, social and time costs of owned and of shared automated vehicle usage in urban and rural areas? (2) How much additional traffic volume would be acceptable from a sustainability perspective when deploying automated vehicles, compared to the usage of human-driven vehicles? (3) How are the costs influenced by different usage characteristics? Overall, our cost-based framework allows us to estimate the sustainability effects of AVs depending on usage characteristics. It consequently extends current academic research and provides practical insights for decision-makers.

Following this introduction, we provide a literature overview related to the characteristics of different types of AV usage. Afterwards, we explain in depth the research approach and the underlying spatial types, selected characteristics and calculations. We then present and discuss the results of our analysis regarding the costs of AV usage. In the final chapter, we conclude and present limitations, further research opportunities and implications.

## Literature review

Previous studies that explore AV usage have examined the effect of introducing such vehicles on VMT by using methods such as transport system simulation and modelling [13–23]. Most of these studies find that AVs are likely to increase VMT, especially for PAVs, and find counterbalancing effects for special cases of SAVs when not only the vehicles, but the rides themselves are shared [2,4]. There are also studies which examine AV energy use and the potential effects of AVs on, e.g., congestion or accidents [24–26]. Through a comprehensive cost model, Letmathe and Paegert [27] investigate the influence of vehicle automation on sustainability from a technological single-vehicle perspective. The present study supplements the previous study by Letmathe and Paegert [27] in two major ways: First, it builds on the established effects of automation and transfers them to AV applications, namely private vehicles and shared vehicles. Second, it focuses on changes in VMT, i.e., rebound effects, whereas the previous study assumed constant VMT. We refer to this previous work for a review of studies related to the external costs and related impacts of automated vehicle technology. In the following, we provide an overview over the body of literature related to the characteristics of different AV usage concepts, including the VMT, number of vehicles, VOT and waiting time.

### Vehicle miles traveled

AVs might affect travel demand due to several effects, thus increasing or decreasing overall mileage. An increased demand could be generated due to more comfortable travel conditions, encouraging passengers to travel more or to switch to AVs from other modes of transport [2]. In addition, AVs enable mobility for additional user groups such as non-driving, elderly or disabled individuals, which alone could increase VMT by up to 14% [28]. On the other hand, ride sharing could bundle individual private vehicle trips and therefore decrease VMT. Ride sharing constitutes a special case of car sharing and means that not only the vehicle, but the ride itself is shared with strangers who have scheduled the same or a similar route. For example, Burghout et al. [18] estimate a VMT reduction of up to 24% if passengers are willing to share rides and accept an up to 50% longer travel time. Moreover, stated preference surveys reveal that AV adoption is influenced by price and individual travel volume, where adoption is encouraged for lower vehicle prices and individuals with high VMT [29]. On the other hand, increased costs due to, e.g., congestion charges, alleviate the choice to use AVs, as Kaddoura et al. [30] demonstrate through simulation experiments.

**Fig 2:**
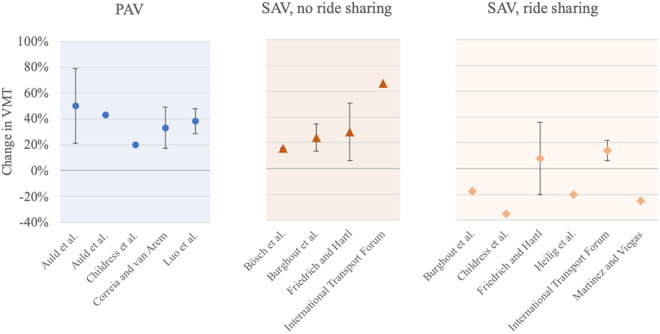
Estimations of the change in VMT for different types of AV usage taken from the literature. The references on the x-axis all consider cases where AVs fully replace human-driven vehicles. The dots mark the mean value of the estimations, and, if a range of estimations is given, it is indicated by a vertical line. References: Auld et al. [14], Auld et al. [15], Bösch et al. [16], Burghout et al. [18], Childress et al. [19], Correia and van Arem [17], Friedrich and Hartl [13], Heilig et al. [20], International Transport Forum [21], Luo et al. [22], Martinez and Viegas [23].

For both PAV and SAV usage, estimations in the literature reveal a big range of potential effects. In Fig 2, we summarize estimated effects from the literature for PAVs, as well as SAVs considered with or without ride sharing. Generally, most of the literature concludes that AVs are likely to increase VMT [4]. Also, PAVs are expected to generate more travel than ride-sharing systems [4], as the latter allow the bundling of traveled routes. Comparing urban and rural areas, automated vehicles are generally expected to generate increased traffic volume in the more dispersed rural areas [31], which points towards the potential rebound effect mentioned previously.

### Number of vehicles

When comparing private car ownership with the usage of shared cars, there is a drastic difference concerning the number of vehicles employed to satisfy travel demand. Whereas privately owned vehicles are parked and unused for more than 23 hours per day [7], a shared vehicle is utilized much more. Thus, the same travel demand can be met by fewer vehicles. Soteropoulos et al. [4] summarize studies from the literature regarding the vehicle reduction potential of automated sharing services, where the number of vehicles needed has a drastic reduction potential of around 90% compared to private cars. For the case of car ownership, Zhang et al. [32] estimate how many households are able to reduce the number of vehicles when they are automated. They show that the number of vehicles can be reduced by 9.5% due to the flexible spatial relocation, which does, however, entail additional empty vehicle mileage.

### Value of time

There are different approaches to estimating the value of time, also referred to as the “value of travel time (savings)”, working with single or differentiated values [33] and using, for instance, revealed preferences, stated preferences or transfer prices [10]. In order to estimate the VOT for AVs in particular, e.g., stated preference questionnaires are applied [3] or AV travel is compared to other modes of transport, such as train rides or conventional taxi services [19,34,35]. The VOT can be differentiated depending on passenger income and trip characteristics [36], usually revealing different costs per time unit for work versus leisure trips.

Table 1 provides an overview of the assumptions and empirical results concerning VOT changes in the context of AV usage, as reported in the literature. Many studies that incorporate VOT make assumptions about the impact of AVs on the VOT, mostly presuming a reduction [14–17,37–41]. A lower VOT reflects the opportunity to use in-vehicle time for other purposes and the associated willingness to accept longer journey times, which can be related to an improved accessibility for AV users [42]. Empirical studies seem to confirm this notion, with effects depending on trip lengths, spatial characteristics, and type of AV deployment [3,43–45]. Zhong et al. [43] make the same differentiation as we do in this paper and consider PAVs and SAVs in urban and rural contexts. They find a user preference for PAVs over SAVs, indicated by a lower VOT for PAVs. Also, they observe that urban AVs offer a higher VOT reduction, likely due to the denser urban traffic.

**Table 1:** Overview of studies related to AV VOT.

Reference	Context	Location	Change in VOT
Auld et al. [14]	Assumptions for PAVs	Chicago, USA	0% to −50%
Auld et al. [15]	Assumptions for PAVs	Chicago, USA	0% to −75%
Bösch et al. [16]	Assumption for SAVs	Zug, Switzerland	0% to −54%
Correia and van Arem [17]	Assumptions for PAVs	Delft, the Netherlands	0% to −50%
Chen and Kockelman [37]	Assumption for SAVs	Simulated region after Austin, Texas	−65%
Cyganski et al. [3]	Empirical results depending on trip length	Brunswick, Germany	−54% to −56%
Gelauff et al. [38]	Assumption for PAVs	The Netherlands	−5% to −20%
Kolarova et al. [45]	Empirical results for commuting trips	Germany	−41%
Kröger et al. [41]	Assumption for PAVs	Germany and USA	0% to −50%
Steck et al. [44]	Empirical results for commuting trips with PAVs/SAVs	Germany	−10% to −31%
Thakur et al. [39]	Assumption for PAVs and SAVs	Melbourne, Australia	−50%
Zhao and Kockelman [40]	Assumption for PAVs and SAVs	Austin, Texas, USA	−25% to −75%
Zhong et al. [43]	Empirical results depending on area type	USA	−8% to −24%

On the other hand, there are also studies arguing that the VOT might not change or increase. Cyganski et al. [46] state that there might be no relevant differences in the use of time when traveling in an AV, as survey respondents anticipate using trips primarily to enjoy the landscape and to talk to other passengers. Explanations for why the VOT might even increase with AVs include longer journeys, which are perceived as more inconvenient [47].

### Waiting time

The travel cost, travel time and waiting time are determining factors for the use of SAVs [48,49]. In the literature, there are different assumptions and results regarding the waiting time for shared vehicles. Burghout et al. [18] regard acceptable waiting times up to 15 minutes. As a lower limit, results in the literature include waiting times of two minutes or less [50]. Table 2 gives an overview of studies related to waiting times for SAV services, which are further described in the following.

**Table 2:** Overview of studies related to SAV waiting times.

Reference	Context	Location	Waiting time [min/trip]
Bischoff and Maciejewski [53]	Simulation results	Rural Switzerland	4.3-5.8
Burghout et al. [18]	Assumption	Urban Sweden (Stockholm)	5-15
Duarte an Ratti [51]	Literature review	Urban USA	3-5
Friedrich and Hartl [13]	Assumption	Urban Germany (Stuttgart)	4
International Transport Forum [21]	Simulation results	Urban Portugal (Lisbon)	3-4
Maciejewski and Bischoff [52]	Simulation results	Urban Germany (Berlin)	2.6-3.5
Viergutz and Schmidt [54]	Simulation results (demand response transport)	Rural Germany (Colditz)	2.7-3.4
Zhang et al. [50]	Simulation results	Urban USA (model city)	1.7

Generally, car sharing in densely populated areas is expected to enable a shorter waiting time than in less densely populated areas [51]. Modelling the city of Lisbon, Portugal, the International Transport Forum observes waiting times of 3–4 minutes for different deployments of car sharing [21]. Studies that focus on Germany or neighboring countries exhibit results in the same order of magnitude. Regarding the Stuttgart region in Germany, Friedrich and Hartl [13] assume a waiting time of 4 minutes until pick up from an SAV service. In a simulation for Berlin, Germany, Maciejewski and Bischoff [52] find passenger waiting times from 2.6 minutes to 3.5 minutes for an automated taxi service fully replacing private cars, depending on the road capacity. For a ride-sharing service supplying rural railway stations in Switzerland, they find average waiting times of 4.3 to 5.8 minutes, depending on whether vehicle rebalancing is applied or not [53]. Investigating a rural German town, the simulations of Viergutz and Schmidt [54] result in a waiting time of 2.7 to 3.4 minutes for a pick-up on-demand transport service.

## Methods and data

**Fig 3:**
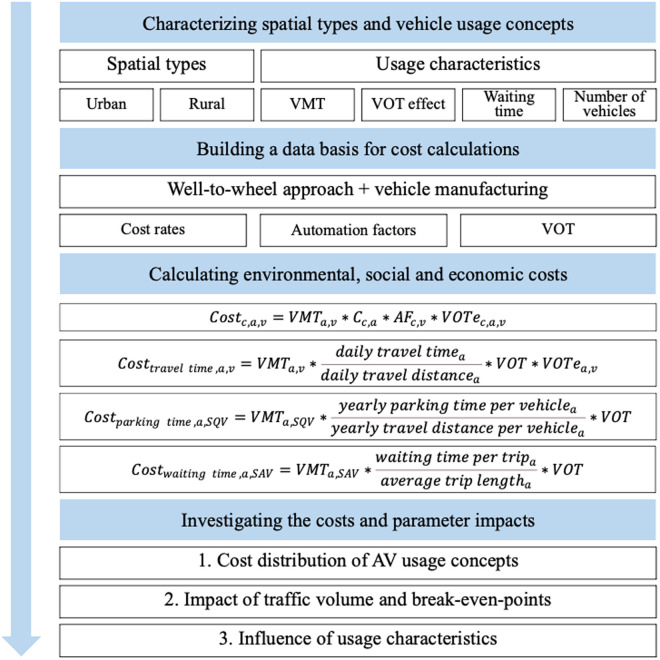
Research approach. For parameter explanations, see equations (1)–(5).

Overall, we regard three usage concepts, each of them based on the deployment of battery electric vehicles: status quo vehicles (SQVs), PAVs and SAVs. Our considerations focus on battery electric vehicles, as it is expected that they will have a rapidly growing share within the vehicle mix, and the European Union plans to allow only zero-emission vehicles from the year 2035 onwards [55]. For the calculations, we use a representative battery electric vehicle, as introduced by Letmathe and Paegert [27]. The research approach followed in this work is illustrated in Fig 3.

We look at the costs of all three concepts in urban and rural areas, respectively, which are described in the following section. We calculate for different usage characteristics (concerning traffic volume, value of time, waiting time and number of vehicles), as described subsequently and summarized in Table 4. Finally, the calculation of the environmental, social, and economic costs is laid out.

**Table 4:** Characteristics of the AV usage concepts.

Usage concept	VOT	Waiting time [min/trip]	Number of vehicles
PAV_u_	−24%	0	−9.5%
SAV_u_	−13%	4	−90%
PAV_r_	−18%	0	−9.5%
SAV_r_	−8%	5	−90%
**Reference**	Zhong et al. [43]	Assumption based on the literature [13,52–54]	Zhang et al. [32], Soteropoulos et al. [4]

### Spatial types

The literature and surveys demonstrate distinct travel patterns in urban and rural areas and regard the implications of areal differences with respect to AVs. Generally, travel destinations are more scattered in rural areas, which are in turn characterized by longer travel distances and greater car usage, with a lower population density at the same time [7,56]. Therefore, any changes in VMT through AVs might be especially impactful in rural areas, as empty vehicle travel is related to longer routes [56]. In urban areas, which are characterized by denser traffic, AVs show a greater influence on the VOT, reflecting a higher perceived liberation from the driving task [43].

We employ the “RegioStaR 7” typology, which is a regional typology for transport research, distinguishing between seven regional types in Germany (Table 3) [57]. The spatial typology was developed by the German Federal Ministry of Transport and Digital Infrastructure, taking into account criteria such as population distribution and journey times [58]. For example, the typology distinguishes between “metropolises” and “regiopolises”, where metropolises have at least half a million inner-city inhabitants or at least one million inhabitants in its surroundings [58]. On the other hand, small town and village areas are characterized by fewer than 20,000 inhabitants and a low population density [58].

**Table 3:** Characteristics of spatial types in Germany. The selected spatial types are highlighted.

Region	Spatial type	Population [million]	Yearly traffic volume of motorized private transport drivers [billion km]	Deviation of traffic volume (of motorized private transport drivers) from population share [%]	Share of motorized private transport in overall traffic volume [%]
Urban	Metropolis	14	83.585	−4.02	59.5
	Regiopolis, large city	12	74.46	−3	67.05
	Medium-sized city, urbanized area	21	168.63	0.73	78.29
	Small-town area, village area	5	48.18	1.43	79.28
Rural	Central city	5	34.31	−0.74	75.29
	Medium-sized city, urbanized area	12	94.9	0.19	79.86
	Small-town area, village area	13	136.145	5.41	84.89
**Total**		82	640.21	0	74.8
**Reference**	Federal Ministry of Transport and Digital Infrastructure [57]	Nobis and Kuhnimhof [7]	Own calculations based on Nobis and Kuhnimhof [60]		

We conduct our main analysis for two selected spatial types in order to show the more extreme poles that represent different mobility requirements of the population. In the S1 Appendix, we present the break-even points of all seven spatial types, demonstrating the robustness of the results with relation to the differentiation between urban and rural regions. The two spatial types for the main analysis were selected as described in the following: Nobis and Kuhnimhof [7] show that the traffic volume of motorized private transport in relation to the population size differs above all for small towns and villages. Thus, we first focus on the small town and village areas in rural regions, which show an increased traffic volume compared to their population share and are clearly distinguishable from medium-sized towns or urban regions (Table 3). Second, we consider large regional cities, where the traffic volume is lower in relation to the population (Table 3). We do not choose metropolitan cities, which constitute a different case characterized by a more pronounced multimodality [7]. This means that in metropolitan cities, cars have a distinctly lower share compared to other modes of transport which we do not focus on here. Most importantly, both of the selected areas are suitable for automated driving solutions, offering opportunities for efficient use in urban areas and improved accessibility compared to established public transport solutions in rural areas [59].

In this work, we refer to the two area types as “rural” and “urban” for illustrative purposes. Together, the selected areas cover 30% of the German population. The results with respect to the other spatial types are presented in the S1 Appendix.

### Usage characteristics

In the following, we describe the regarded characteristics of the AV usage concepts, concerning VMT, the number of vehicles, VOT and the waiting time. The usage characteristics constitute the factors that the calculations depend on, as described below.

With regard to the 2x2 study design, we consider four concepts of AV usage: “urban PAVs” (PAV_u_), “rural PAVs” (PAV_r_), “urban SAVs” (SAV_u_) and “rural SAVs” (SAV_r_). The PAV concepts refer to privately owned AVs. For a SAV, we refer to a publicly shared vehicle with flexible pick-up and drop-off possibilities, which is not owned by the passenger. Each concept is characterized by different VOTs, waiting times and fleet sizes, which are analyzed in terms of their respective impacts on the results. To begin with, we assume that all vehicles are occupied by one individual. In addition, we subsequently discuss the sustainability impact and the time-related impact of vehicle occupancy. AV usage is compared to using “urban status quo vehicles” (SQV_u_) or “rural status quo vehicles” (SQV_r_), which are based on human-driven vehicles with current traffic volume.

As the VMT impact of AVs is highly uncertain, we include VMT as an independent variable in the calculation. In this way, we determine the costs of AV usage as a function of VMT (Fig 6) and can determine the break-even points of the different AV concepts with the SQVs (Fig 7). When comparing the usage concepts, the notion of higher VMT for PAVs and rural areas should be taken into account (see Fig 2).

For the main analysis, we employ the characteristics summarized in Table 4, specifying the VOT, the waiting time and the number of vehicles for each AV usage concept. First, based on the VOT of conventional vehicles, we adopt VOT changes for the AVs. Concerning changes in VOT, we employ the values from Zhong et al. [43], which represent empirical results corresponding to the considered usage concepts of PAV_u_, PAV_r_, SAV_u_ and SAV_r_. For instance, the VOT for PAV_u_ is influenced the most, as vehicle automation can likely offer a liberation from driving in dense traffic situations [43]. Since this study addresses the same usage concept as our work and we are not aware of any other research that differentiates between differences in both ownership and spatial types, we adopt the values for our calculations. Second, we assume a waiting time of four minutes in urban areas [13,52] and five minutes in rural areas [53,54], which corresponds to previous literature related to SAV deployment in Germany and Switzerland. The distinguishment also represents the notion that waiting times are lower in urban areas, which are more dense than rural areas [51]. Third, AVs are shown to be able to meet travel demand with fewer vehicles. AVs can be scheduled in such a way that the number of private vehicles per household can be reduced at the expense of more VMT through empty vehicle travel [32]. Regarding SAVs, Soteropoulos et al. [4] review studies from different geographical contexts and conclude that travel demand could be served with substantially fewer vehicles, as vehicles are not owned and not parked most of the time, but can be deployed flexibly [4]. Regarding PAVs, we are not aware of any studies that quantify the number of necessary vehicles specifically for Germany. In order to include the effects from vehicle relocation, we therefore transfer the results from Zhang et al. [32] to our study. Due to the present uncertainties, we later also show how the results are affected when varying the usage characteristics (Fig 8).

### External costs

In order to evaluate the sustainability impact of vehicles, the methodology of life-cycle assessment can be used to compare different usage concepts with regard to different life-cycle phases, e.g., manufacturing, usage or recycling [61]. A well-to-wheel approach is often employed in transport research, which includes vehicle usage itself (tank-to-wheel) as well as the energy supply (well-to-tank) [62]. Our framework encompasses this well-to-wheel perspective and additionally includes vehicle manufacturing, which is important to consider when comparing car ownership and car sharing, as the latter is expected to require fewer vehicles. We present the results in monetary terms, which enables an easy comparison and is supported by the methodological conventions of the European Commission and the German Environment Agency [8,9].

External costs emerge for the environment and society whenever costs are not borne by their originator or by the user of a product [63,64]. Following Matthey and Bünger [9] as well as Letmathe and Paegert [27], we choose the year 2020 as the reference year for the cost assessment, facilitating a comparison with prior results. Using the consumer price index [65], we adjust any cost rates from sources which refer to years other than 2020. Note that shifting costs to years other than 2020 would indeed change the level of costs but not the overall relation of cost components to each other.

Most of the cost components vary locally. In urban areas, for instance, noise emissions affect more people, resulting in greater harm and, consequently, in higher external costs. Therefore, the cost rates differ between urban and rural areas for components that depend on local circumstances. In our model, this affects all components except the costs of vehicle manufacturing and the costs of the energy supply. The cost rates we use for urban and rural areas are listed in Table 5.

**Table 5:** Cost rates for the status quo vehicle in urban and rural areas. The cost rates are adjusted to the year 2020. Units: ct = euro cent, vkm = vehicle kilometer, h = hour.

Area	Component	Unit	Cost rate	Reference
Urban and rural	Manufacturing	ct/vkm	3.38	Matthey and Bünger [9]
Urban and rural	Energy supply	ct/vkm	1.68	Letmathe and Paegert [27]
Urban	PM (local)	ct/vkm	0.59	Letmathe and Paegert [27]
Rural	PM (local)	ct/vkm	0.09	Own calculations based on Letmathe and Paegert [27], executed with rural cost rates from Matthey and Bünger [9]
Urban	Noise	ct/vkm	1.91	Letmathe and Paegert [27]
Rural	Noise	ct/vkm	0.02	Own calculations based on Letmathe and Paegert [27], executed with rural cost rates from Jochem et al. [66] and van Essen et al. [67]
Urban	Congestion	ct/vkm	8.94	Letmathe and Paegert [27]
Rural	Congestion	ct/vkm	5.52	Own calculations based on Letmathe and Paegert [27] with an average time of 40 hours [68] lost in congestion, executed with rural cost rates from van Essen et al. [8]
Urban	Accidents	ct/vkm	4.37	van Essen et al. [69]
Rural	Accidents	ct/vkm	1.62	van Essen et al. [69]
Urban and rural	Time (business)	€/h	17.26	van Essen et al. [8]

Overall, the employed cost rates represent the average conditions in urban or rural areas of Germany. For example, the cost rate for local PM considers the amount of vehicle emissions from abrasion and resuspension [70], as laid out in previous research [27]. The quantities are then multiplied with the emission cost rates from the German Environment Agency [9], which vary for urban and rural areas. Again, this is due to the fact that population density differs and therefore distinct numbers of people are affected by the emissions. Related to the different components, we refer to the references stated in Table 5, which lay out the background and calculation of the cost rates in depth.

### Calculation of environmental and social costs

The environmental and social pillars of the cost model represent externalities which arise from vehicle usage and production. The SQV costs are calculated based on the cost rates and the traffic volumes within urban and rural areas (Table 3). Additionally, we apply automation factors for AV usage, taking into account effects such as an improved traffic flow or expected higher road safety [27] (Table 6).

**Table 6:** Applied automation factors.

Component	AV usage concept	Automation factor	Reference
Manufacturing	PAV	90.5%	Zhang et al. [32]
Manufacturing	SAV	10%	Soteropoulos et al. [4]
Energy supply	PAV, SAV	82.7%	Letmathe and Paegert [27]
PM (local)	PAV, SAV	87.6%	Letmathe and Paegert [27]
Congestion	PAV, SAV	28.25%	Letmathe and Paegert [27]
Accidents	PAV, SAV	50%	Letmathe and Paegert [27]

Regarding the congestion costs of AV usage, we also incorporate the effect of a changed VOT. For example, if the VOT in automated vehicles is 10% lower or higher, we also assume 10% lower or higher congestion costs for AVs than for conventional vehicles. Again, this relates to the notion that spending time in an AV might be perceived as more comfortable or less comfortable due to the above-mentioned reasons. Thus, overall, congestion costs of AVs differ from the SQV costs in two ways: Primarily, an improved traffic flow through AV technology and secondly, a change in the VOT [27]. Equation (1) shows how the environmental and social costs are calculated.


$${Cost}_{c,a,v}={VMT}_{a,v}*C_{c,a}*{AF}_{c,v}*{VOTe}_{c,a,v}$$ (1)

${Cost}_{c,a,v}$ Cost of a component c for an area a and vehicle usage concept v

C Set of environmental and social components with C={manufacturing,energysupply,PM(local),noise,congestion,accidents}

A Set of areas with A={urban,rural}

V Set of vehicle usage concepts with V={SQV,PAV,SAV}

${VMT}_{a,v}$ Amount of traffic volume of all vehicles from usage concept v within an area a per year, which is employed as an independent variable. The status quo traffic volumes are denoted in Table 3.

$C_{c,a}$ Cost rate for component c and area a (Table 5)

${AF}_{c,v}$ Automation factor for a component c and vehicle usage concept v (Table 6), note that ${AF}_{c,SQV}=1\forall c\in C,{AF}_{noise,v}=1\forall v\in V$)

${VOTe}_{c,a,v}$ Effect arising from a change in VOT (derived from Table 4), which concerns the congestion costs. ${VOTe}_{c,a,v}=1\forall c\in C\setminus \left\{congestion\right\}$, and ${VOTe}_{c,a,SQV}=1\forall a\in A,c\in C$.

Previous research [27] has estimated the upper and lower bounds of the external costs related to vehicle automation and other future developments such as a higher share of renewable energies. In this research, we focus on the main automation factors and keep a focus on the different usage concepts and area types. In other words, we refrain from incorporating the upper and lower bounds of such future developments since these have been laid in previous work.

### Calculation of time costs

In the economic pillar, we include time cost in terms of travel time costs, parking time costs and waiting time costs. The time costs calculated in this paper constitute the aggregated generalized costs arising due to the yearly traffic volume within the urban or rural areas. At an individual level, time costs vary according to personal circumstances, i.e., income. This level of individual differentiation, however, is not part of the present work. For time costs, we only consider business trips, which account for 21% of all trips in both urban and rural areas [60]. We do not include private leisure trips, for which Kolarova et al. [45] find no change in the VOT for AVs compared to conventional vehicles. For the calculations, we employ the German VOT for business trips, as provided by the European Commission [8] (Table 5). This results in a VOT of 3.62 €/h (equation (2)).


$$VOT=Shareofbusinesstrips*{VOT}_{businesstrips}=21\;\%*17.26\frac{€}{h}=3.62\frac{€}{h}$$ (2)

VOT Value of time employed for the main analysis

Shareofbusinesstrips Proportion of business trips among all trips, employed from the online tool of the study “Mobility in Germany” [60]

${VOT}_{businesstrips}$ Value of time for business trips (Table 5)

First, we estimate the travel time costs for each of the usage concepts, i.e., the value of the time which is spent in the vehicle. For these and the other time costs, we employ the travel times and travel distances for both urban and rural areas from the study “Mobility in Germany” and its respective online tool commissioned by the German Federal Ministry of Transport and Digital Infrastructure [7,60]. Equation (3) illustrates how the travel time costs are calculated for the usage concepts. Please find the explanations of previously mentioned parameters with the equations above. The formula calculates the travel time costs that arise in an area per year for the different vehicle concepts, respectively. In order to relate the arising VMT to the VOT, which are expressed in kilometers and €/h, the travel time and travel distance must be included in order to obtain the total travel time costs related to the occurring traffic.


$${Cost}_{traveltime,a,v}={VMT}_{a,v}*\frac{{dailytraveltime}_{a}}{{dailytraveldistance}_{a}}*VOT*{VOTe}_{a,v}$$ (3)

${Cost}_{traveltime,a,v}$ Travel time cost for an area a and vehicle usage concept v.

${dailytraveltime}_{a}$ Daily vehicle travel time for an area a, employed from the online tool of the study “Mobility in Germany” [60]

${dailytraveldistance}_{a}$ Daily travel distance for an area a, employed from the study “Mobility in Germany” [7]

${VOTe}_{a,v}$ Effect arising from a change in VOT for an area a and vehicle usage concept v (derived from Table 4, note that ${VOTe}_{a,SQV}=1\forall a\in A$)

Second, we incorporate parking times for the SQVs. Regarding AV usage, we expect the vehicle to park itself, so that the users do not have to spend time parking. Here, we assume a per-vehicle parking time of 57 hours for urban areas and an average 41 hours yearly for rural areas [71]. Equation (4) shows how the parking time costs of the SQV_u_ and SQV_r_ are calculated. In this context, the parking time and travel distance per vehicle are related to the overall VMT within an area. The evaluation of the parking time with the VOT then results in the total parking time costs.


$${Cost}_{parkingtime,a,SQV}={VMT}_{a,SQV}*\frac{{yearlyparkingtimepervehicle}_{a}}{{yearlytraveldistancepervehicle}_{a}}*VOT$$ (4)

${Cost}_{parkingtime,a,SQV}$ Parking time cost for a SQV in an area a

${yearlyparkingtimepervehicle}_{a}$ Yearly parking time for an area a, employed from the literature [71]

${yearlytraveldistancepervehicle}_{a}$ Yearly travel distance for an area a, employed from the study “Mobility in Germany” [7]

Third, waiting time for vehicle arrival emerges for the SAVs, whereas the SQVs and PAVs do not require any waiting. We employ waiting times of four minutes for urban areas and five minutes for rural areas, as described previously. Consequently, the waiting time costs for SAVs are calculated in accordance with equation (5).


$${Cost}_{waitingtime,a,SAV}={VMT}_{a,SAV}*\frac{{waitingtimepertrip}_{a}}{{averagetriplength}_{a}}*VOT$$ (5)

${Cost}_{waitingtime,a,SAV}$ Waiting time cost for an area a related to SAV usage

${waitingtimepertrip}_{a}$ Waiting time per trip, with ${waitingtimepertrip}_{urban}=4min$, ${waitingtimepertrip}_{rural}=5min$

${averagetriplength}_{a}$ Length of an average trip in area a, employed from the study “Mobility in Germany” [7]. Waiting time emerges per trip.

## Results and discussion

### Distribution and amount of costs

**Fig 4:**
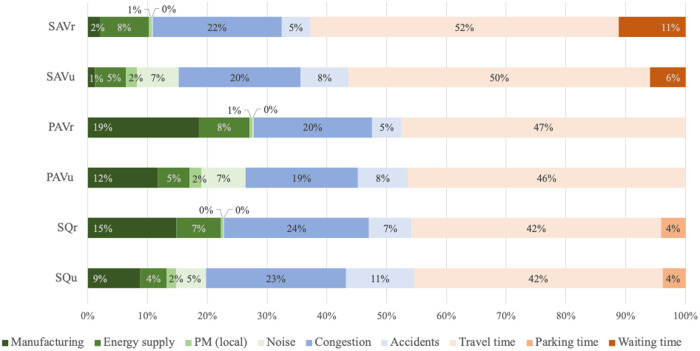
Distribution of costs for the vehicle usage concepts.

The distribution of environmental costs is different for urban and rural areas (Fig 4). Generally, local PM and noise emissions are much more dominant in urban areas because of the denser population and thus higher exposure. However, in rural areas, the costs of vehicle manufacturing and energy supply emissions have a larger share. This is because rural areas are generally characterized by higher VMT, which translates into a need for a greater number of vehicles and higher energy use.

The social costs of congestion and accidents together comprise about a quarter to a third of the total costs. For PAV_r_, for example, social costs account for only 25% of total costs due to the dominance of time costs.

Time costs constitute the largest amount, with travel time costs amounting to 42–52% of overall costs, and parking time to 4% of SQV costs. Waiting time costs are relevant for the SAVs, accounting for 6% of costs in urban areas and 11% in rural areas. This is not only due to the assumption that waiting times are higher in rural areas than in more densely populated urban areas. Urban areas are characterized by a lower yearly traffic volume as well as by shorter trips on average. In rural areas, while traffic volume is higher and trips are longer, the higher traffic volume dominates, resulting in a higher number of trips made within rural areas compared to urban areas. Consequently, as waiting time emerges per trip, the sum of waiting time in rural areas is higher. Naturally, the results are bound to the usage characteristics of the PAVs and SAVs, e.g., the presumed waiting time. After presenting the results of the main analysis, we show how the costs are influenced by the model assumptions related to the waiting time, VOT and the number of necessary vehicles in order to serve the travel demand. For example, we show how an increase (decrease) of waiting time affects total costs, which would consequently increase (decrease) the share of time costs presented here.

**Fig 5:**
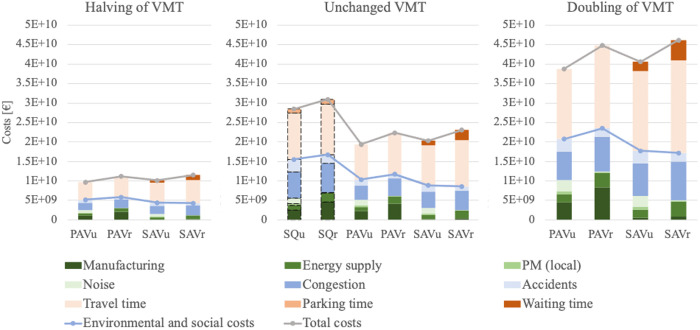
Costs of AV usage for halved, unchanged and doubled VMT. SQV_u_ and SQV_r_ serve as a baseline and are calculated based on unchanged, i.e., current, VMT.

In Fig 5, we exemplarily present the costs of AV usage for a halving of VMT, for unchanged VMT and for a doubling of VMT compared to the usage cases of SQV_u_ and SQV_r_, respectively. The SQV costs are used as a baseline and amount to a total of 28.48 billion € for urban areas and 30.96 billion € for rural areas. The costs of the AVs change depending on VMT, with PAVs being primarily influenced by environmental and social costs, while SAVs, particularly the SAV_r_, are predominantly affected by time costs.

### Costs as a function of VMT

**Fig 6:**
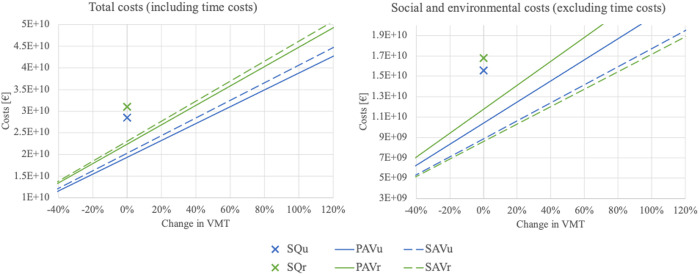
Costs as a function of VMT with and without time costs.

Comparing PAVs to SAVs, Fig 5 shows that the benefits vary depending on whether time costs are considered or not. Fig 6 reports the sum of costs both with and without time costs as a function of VMT and allows a more in-depth analysis. Here, a change in VMT of 0% means unchanged traffic volume, a 100% change in VMT corresponds to a doubling of traffic volume, et cetera.

Regarding total costs (including time costs), PAVs result in lower costs for the same VMT than SAVs do for both urban and rural areas. However, this result changes when disregarding time costs and focusing only on the sum of social and environmental costs: The social and environmental costs diverge more regarding PAVs and SAVs, with the SAVs being more beneficial.

The effect of time costs is particularly strong for SAV_r_. For the same VMT, we show that SAV_r_ is the least favorable option when considering total costs but the most favorable option when excluding the personal time costs and focusing only on social and environmental costs. This creates a dichotomy: SAV_r_ is the most environmentally and socially beneficial option but the least attractive one when the time value of passengers is taken into account. When interpreting the results, it should be noted that PAVs and SAVs are expected to generate differing VMT, with PAVs likely to generate more traffic than, e.g., ride-sharing AVs [4]. In addition, AV usage in rural areas may generate more VMT than in urban areas because journeys are more dispersed [31]. Consequently, PAV_r_ constitute the usage case where the most additional traffic is likely to be generated. However, in terms of social and environmental costs, PAV_r_ exhibit higher costs than any other concept, regardless of VMT, which can be seen as another critical issue.

**Fig 7:**
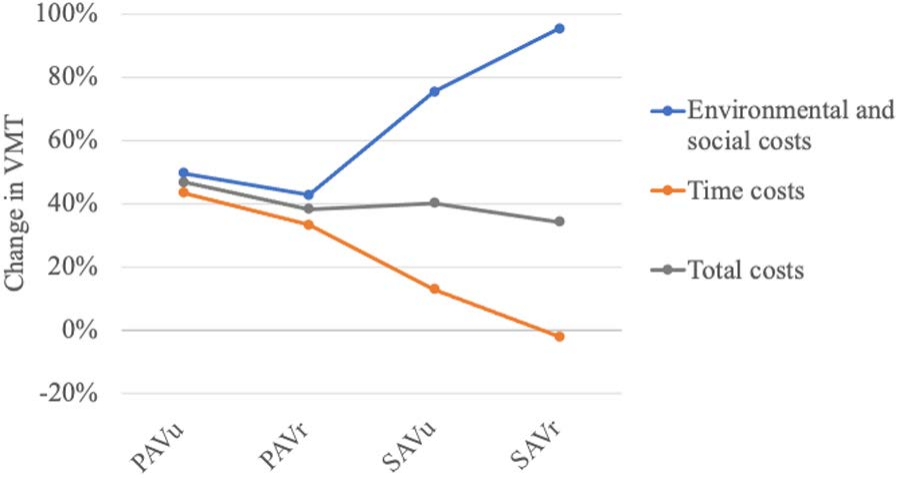
Break-even points of AV usage with the status quo.

The main objective of this work is to quantify the extent of additionally generated traffic stemming from AVs that will not lead to higher environmental, social and economic costs. Fig 7 shows the amount of additional or reduced VMT that breaks even with the status quo when replacing conventional individual transport with each AV usage concept.

The break-even points are analyzed for the sum of environmental and social costs, the sum of time costs and the total sum of costs. For PAVs in rural and urban areas, the break-even points lie relatively close together. For environmental and social costs, 50% of additional VMT are acceptable for PAV_u_ and, at the lower end, up to 43% for PAV_r_. Considering time costs, the break-even points shift downwards and lie at 44% additional VMT for PAV_u_ and at 33% for PAV_r_.

For SAVs, the break-even points for environmental and social costs drift apart compared to time costs, displaying again the dichotomy of the different perspectives. Regarding environmental and social costs, SAVs allow an additional VMT of 75% for urban areas and 95% for rural areas. The time costs for SAV_u_, however, break even at an additional VMT of 13%. For SAV_r_, VMT would actually have to decrease by 2% to not be detrimental to time costs compared to the status quo. In this case, the comfort of AVs does not outweigh the waiting time costs that are incurred.

In terms of total costs, the social, environmental and time effects overlap. Overall, the PAV_u_ allow for 47% additional VMT, while for SAV_r_ only up to 34% additional VMT would not lead to higher total costs compared to the status quo.

### Influence of usage characteristics

**Fig 8:**
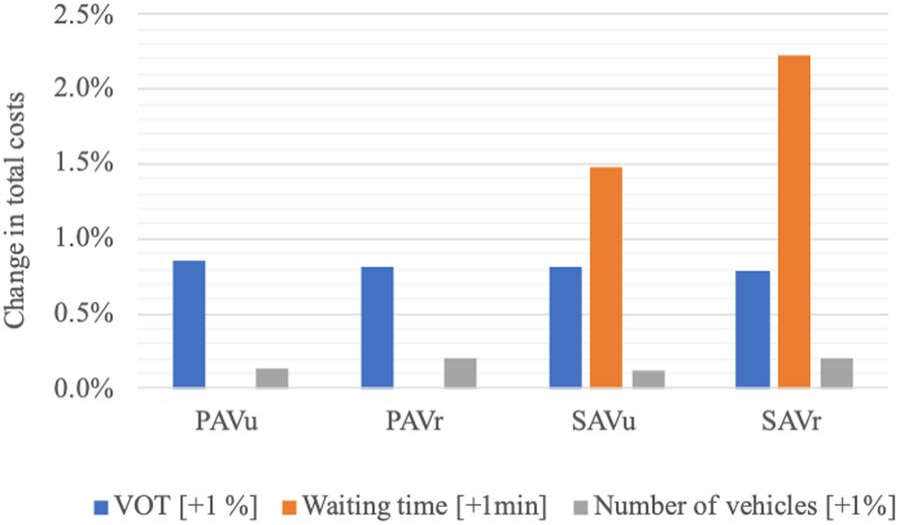
Change in total costs in relation to the usage characteristics (Table 4). Example: If waiting time increases by 1 min, the total costs of SAV_u_ rise by 1.48%.

In the following, we regard the variation of total costs with respect to a change in the usage characteristics reported in Table 4, namely VOT, waiting time and number of vehicles (Fig 8). Besides VMT, these are the characteristics which influence the amount of environmental, social and economic costs, with differing levels of influence as reported below.

First, the influence of the VOT on total costs is similar across all AV usage concepts, where the total costs rise by around 0.8% for an increase in the VOT by one percentage point.

Second, waiting time only occurs with car sharing. Consistent with previous results, waiting time has a strong influence, especially in rural areas, where a one-minute increase in waiting time corresponds to a 2.2% increase in total costs. Depending on the passenger behavior as well as on the waiting situation, however, waiting time might not be perceived by passengers as such. For example, in the case of an at-home pick-up, passengers might be able to use some or all of the waiting time for other (productive) purposes. This should be taken into account when interpreting the results at an individual level.

Third, the number of vehicles needed for each of the AV usage concepts also has an impact on total costs. In rural areas, the impact is more pronounced due to the higher traffic volume compared to urban areas, i.e., rural areas require a greater number of vehicles to meet the demand. For the PAV_r_ and SAV_r_, an increase in the number of vehicles by one percentage point leads to an increase of total costs by 0.21% and 0.2%, respectively. For the PAV_u_ and SAV_u_, total costs increase by 0.13% and 0.12%, respectively.

Overall, this analysis shows that the different AV usage concepts allow a higher traffic volume up to a break-even point, until which they will lower current environmental, social and economic costs. The costs are sensitive to the characteristics of AV usage, in particular the amount of waiting time for car sharing.

### Vehicle occupancy and additional passenger VMT

For the cost assessment, we have focused on one driver for the SQV or one passenger for the AV usage so far. Naturally, the vehicle occupancy rate influences the amount of overall environmental, social and economic costs in different ways, depending on the perspective taken. From a per-passenger perspective, the environmental and social costs benefit from a high vehicle occupancy rate. For example, two persons traveling to the workplace in one vehicle instead of in two vehicles only need half of the energy supply per passenger (when disregarding any effect of the passenger weight). From a vehicle perspective, though, time costs increase with the number of passengers per vehicle, as time costs occur for each passenger. In this study, we have calculated the time costs of SQV, PAV and SAV usage for traffic occurring in urban and rural areas, employing the average costs. On an individual level, however, time costs differ, e.g., based on personal income [36]. With regard to the use of AVs, the time value could change differently for former drivers and former passengers. There is, however, a lack of empirical studies. In this vein, Zhong et al. [43] only find insignificant results for passengers and can thus not estimate a VOT.

In this research, the costs for entire urban and rural areas are determined. For the SQVs, the results are based on the current VMT of motorized private transport drivers [7]. As an extension to the results, we examine how total costs change when considering additional passengers. For this purpose, we consider the VMT of motorized private transport passengers [7] as an addition to the time costs. In the absence of other information, we assume the same VOT and VOT reduction as for the first user.

**Fig 9:**
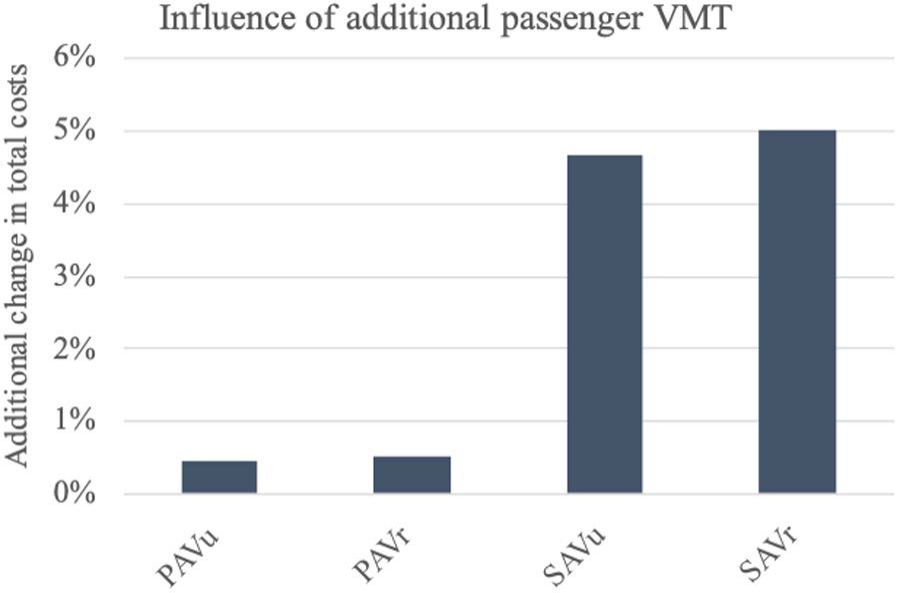
Change in total costs when considering the VMT from additional motorized private transport passengers. The change in total costs of AV usage already takes into account (subtracts) the change in status quo costs.

Fig 9 shows that the costs of SAVs are influenced more by the additional passenger VMT, since car sharing costs are more strongly influenced by time costs, e.g., waiting time. The costs of PAV_u_ and PAV_r_ increase by 0.4% and 0.5%, respectively, in addition to the rise of the status quo costs. In contrast, the costs of SAV_u_ and SAV_r_ increase by 4.7% and 5%, respectively, again in addition to the now also higher respective status quo costs. This analysis once more shows the role of time costs as a critical factor among the considered components of the framework.

In general, a high vehicle occupancy through ride sharing should be encouraged, as it can decrease VMT [4]. However, shared AVs have a less positive effect on the VOT than non-shared AVs [43] and are therefore unlikely to be the preferred option of passengers.

### Accounting for leisure trip VOT

**Fig 10:**
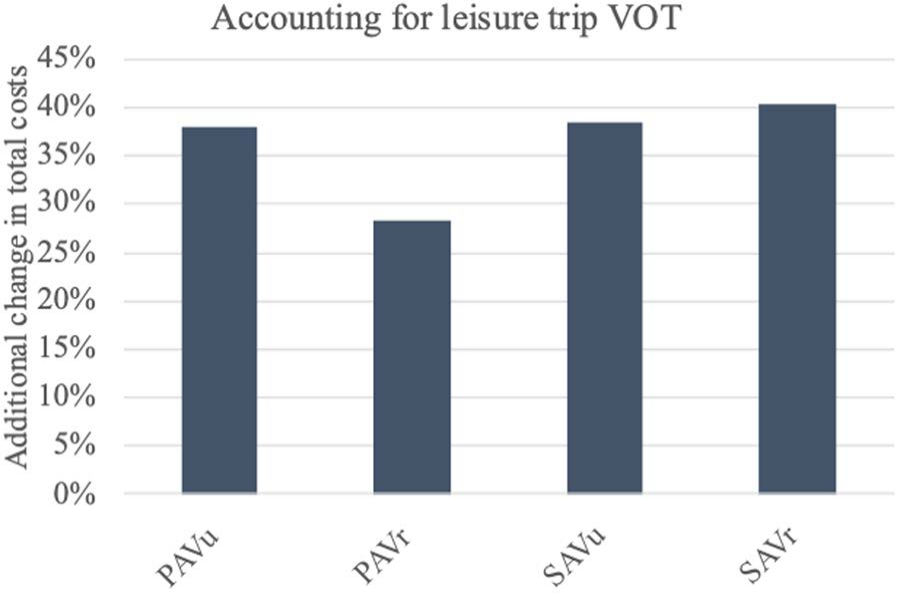
Change in total costs when accounting for leisure trip VOT. The change in total costs of AV usage already takes into account (subtracts) the change in status quo costs.

In addition to the base model, we calculated the results when the time costs for leisure trips are included. In relation to equation (2), this means that we added supplementary costs of 8 €/h [8] for 79% of trips [60] to account for leisure journeys. We apply the same VOT to all leisure trips with SQVs, PAVs and SAVs, adopting equal VOT for all vehicle types [45]. Correspondingly, the additional change in total costs for the different AVs ranges from 29% for the PAV_r_ to 40.4% for the SAV_r_ (Fig 10).

This is largely due to the increase in individual time costs, whereas the environmental and social costs are affected only through the additional congestion costs for leisure trips. Overall, SAVs are affected more than PAVs since waiting time emerges for leisure trips, whereas no waiting time arises when using PAVs. Also, the valuation of travel time in urban areas is more affected than in rural areas. This is because the VOT for leisure trips has a larger difference to the VOT for business trips in urban areas. In urban areas, commuters perceive AV usage as more convenient than in rural areas due to the traffic situation [43], i.e., the VOT for business trips is lower. While this results in larger additional costs changes for the PAV_u_ in comparison to the PAV_r_, the effect is offset for SAVs by the waiting time costs.

Naturally, the time costs are subject to the VOT, which again illustrates the contrast between the benefits to society (environmental and social costs) and the benefits to the individual (time costs), which need to be evaluated separately since they represent different perspectives and are clearly influenced by different factors. As shown here, VOT considerations largely influence individual time costs and thus the attractiveness of usage from the individual passenger’s perspective.

## Conclusion

The results show the environmental, social and economic costs of PAV and SAV usage, respectively, in urban and rural areas compared to status quo conditions. We show that, overall, an additional traffic volume of between 34% and 47% could be accepted from a sustainability perspective, depending on the usage concept. Rural car-sharing constitutes the most distinct case, where the environmental and social pillars exhibit the lowest costs across all AV usage concepts, but where the time costs are the highest. This means that in rural areas, car sharing benefits the environment and society the most, but it is also the least attractive due to the time costs, which reflect comfortability and waiting, posing a dichotomy. Overall, the costs depend on VMT, as well as on other characteristics (value of time, waiting time, number of vehicles), where the SAV costs are particularly sensitive to variations in waiting time.

### Limitations and further research

The present research could be extended in different directions. Here, we have considered urban and rural areas in Germany with their aggregated characteristics. Future research could extend this analysis by comparing costs at a local level, focusing on differences within urban areas or within rural areas themselves. The developed cost framework could also be applied with regards to other countries, which was outside the scope of the present work. Furthermore, user groups could be diversified according to their characteristics, such as varying VOTs.

The framework focuses on costs which reflect the effects that arise for the environment, for society and for the passengers’ use of time. Besides the regarded components, there are other factors that are likely influenced by AV deployment, e.g., parking spaces, urban sprawl, and access to mobility for disabled individuals [12]. Due to the non-availability of sufficient data or the qualitative nature of some aspects we were not able to integrate such factors into our model. Additionally, there are internal tangible monetary factors, e.g., fuel costs and driving fees, which could be combined with the beforementioned values to further compare the costs from a user perspective.

This study regards the isolated deployment of highly automated passenger cars. It does not consider the effect of AV usage on transport mode choice and the resulting external effects on the transport sector as a whole. Employing the present framework, quantifying the interaction effects of different modes, such as public transport with AVs, would provide more insights regarding the sustainability of the whole passenger transport sector. This would allow potential indirect rebound effects to be included, i.e., an increased usage of other transport modes.

### Implications

This study contributes to the quantitative understanding of the dual nature of AV deployment effects on the sustainability of motorized individual transport. Considering the environmental, social and economic components, AV usage is beneficial compared to SQV usage up to a level of additionally generated traffic of 34–47%, depending on the area and type of usage (car ownership versus car sharing), which leaves some room for rebound effects. Nonetheless, policymakers and system providers should strive to keep future traffic volume below this threshold and, generally, as low as possible.

The results concerning the different vehicle concepts need to be classified against the background of the existing literature [13–23] with regard to potential VMT changes. Accordingly, Table 7 classifies the break-even points calculated in this study, showing which usage concepts are particularly vulnerable to rebound effects.

**Table 7:** Classification of calculated break-even points related to VMT changes.

Usage concept	Break-even-point			Potential VMT change	Severity of potential rebound effect		
	Total	Environmental and social	Time		Total	Environmental and social	Time
PAV_u_	47%	50%	44%	37%	tolerable	tolerable	tolerable
PAV_r_	38%	43%	33%		tolerable	tolerable	critical
SAV_u_	40%	75%	13%	8%, −13% (ridesharing)	tolerable	tolerable	tolerable
SAV_r_	34%	96%	−2.1%		tolerable	tolerable	critical
**Reference**	Own calculations (Fig 7)			Mean based on the literature [13–23] (Fig 2)	Own interpretation: tolerable if potential VMT change < break-even point		

The results show that AV use is potentially critical in rural areas with regard to time costs, especially for SAVs when only the vehicles are shared but not the rides. This implies that SAVs in rural areas are less attractive for potential users, even though they would tolerate the largest rebound effect concerning the environmental and social costs. Overall, SAVs are more beneficial than PAVs with respect to environmental and social costs. Therefore, sharing concepts should be made more attractive to passengers: From an operator’s perspective, AV sharing should offer short waiting times. From a policy-making perspective, SAVs could be subsidized to encourage their use and to reap the positive externalities as perceived economic rewards are shown to positively impact the intention to use car sharing systems [72]. Other research has shown that congestion fees discourage private vehicle usage [36] and that taxing empty vehicle travel reduces traffic [25], demonstrating that internalization policies are effective in reducing negative environmental and social impacts.

## Supporting information

S1 Appendix Break-even points of spatial types. (PDF)
