# Supplementary material for: Automated vehicles and sustainability when considering rebound effects
Source: PLoS One. 2025 Aug 1;20(8):e0329193. doi: 10.1371/journal.pone.0329193 (PMC12316294; doi:10.1371/journal.pone.0329193)
Supplement: S1 Appendix — (PDF) [file pone.0329193.s001.pdf]

Appendix

Break-even points of spatial types

Table 1: Break-even points of additional traffic volume from AV applications in relation to the spatial types. The break-even point states how much additional traffic volume could be endured to not worsen current status quo conditions.

		PAV			SAV		
**Region**	**Spatial type** [1]	**Environmental and social costs**	**Time costs**	**Total costs**	**Environmental and social costs**	**Time costs**	**Total costs**
Urban	Metropolis	49.7%	43.6%	46.7%	75.4%	15.0%	40.7%
	Regiopolis, large city	49.7%	43.5%	46.8%	75.4%	12.9%	40.2%
	Medium-sized city, urbanized area	49.7%	46.4%	48.4%	75.4%	11.8%	43.1%
	Small-town area, village area	49.7%	46.0%	48.3%	75.4%	11.7%	43.9%
	Medium weighted by traffic volume	*49.7%*	*45.1%*	*47.7%*	*75.4%*	*12.7%*	*42.1%*
Rural	Central city	42.8%	31.3%	36.5%	95.5%	0.0%	30.1%
	Medium-sized city, urbanized area	42.8%	30.9%	36.2%	95.5%	-0.9%	28.7%
	Small-town area, village area	42.8%	33.3%	38.3%	95.5%	-2.1%	34.2%
	Medium weighted by traffic volume	*42.8%*	*32.2%*	*37.3%*	*95.5%*	*-1.4%*	*31.7%*

Overall, the break-even points of urban and rural regions exhibit different patterns corresponding to the main analysis. Shared automated vehicles (SAVs) in rural regions allow for the largest amount of additional traffic volume when considering the environmental and social costs. Contrastingly, though, they do not allow for additional traffic volume when considering time costs. This dichotomy can be observed in all rural regions, showing that shared vehicles in rural areas are the most advantageous from the perspective of the environment and society, but the least attractive with regards to individual time costs.

The break-even point with relation to the environmental and social costs does not vary between spatial types within one region, since we apply the same cost rates for either urban or rural contexts. Subsequent research could further differentiate the costs with relation to specific cities or villages in urban and rural regions, respectively.

References

1. Federal Ministry of Transport and Digital Infrastructure. RegioStaR Regional Statistical Spatial Typology for Mobility and Transport Research. Bonn; 2021.
